# Human Rabies in the WHO Southeast Asia Region: Forward Steps for Elimination

**DOI:** 10.4061/2011/383870

**Published:** 2011-09-21

**Authors:** Gyanendra Gongal, Alice E. Wright

**Affiliations:** Disease Surveillance and Epidemiology, WHO Regional Office for South East Asia, New Delhi 110002, India

## Abstract

There are eleven Member States in the WHO southeast Asia region (Bangladesh, Bhutan, Democratic People's Republic of Korea, India, Indonesia, Maldives, Myanmar, Nepal, Sri Lanka, Thailand, Timor-Leste) of which eight are endemic for rabies. More than 1.4 billion people in the Region are at risk of rabies infection, and approximately 45% of worldwide rabies deaths occur in Asia. Dog bites account for 96% of human rabies cases. Progress in preventing human rabies through control of the disease in dogs has been slow due to various factors. Innovative control tools and techniques have been developed and standardized in recent years. The introduction of cost-effective intradermal rabies vaccination regimens in Asian countries has increased the availability and affordability of postexposure prophylaxis. Elimination of rabies is not possible without regional and intersectoral cooperation. Considering the importance of consolidating achievements in rabies control in Member countries, the WHO Regional Office for southeast Asia has developed a regional strategy for elimination of human rabies transmitted by dogs in the Region. They have committed to provide technical leadership, to advocate national health authorities to develop major stakeholder consensus for a comprehensive rabies elimination programme, and to implement national strategies for elimination of human rabies.

## 1. Introduction

Rabies is an ancient viral zoonotic disease that is invariably fatal in humans and mammals. The disease circulates in two epidemiological cycles: an urban cycle involving maintenance of infection in dog populations and a sylvatic cycle involving wildlife. There is a possibility of spill-over of rabies virus from dogs to wildlife and vice versa. 

Dogs are the most important rabies reservoir. Human cases have also been reported due to exposure to rabid cats and wildlife. Mongoose (*Herpestes *spp.), jackals (*Canis aureus*), foxes (*Vulpes bengalensis*) and wolves (*Canis lupus*) have been incriminated as wildlife reservoirs of rabies in Bangladesh, India, and Nepal [1]. Recent studies on the *Nepalese* field rabies virus indicate that it belongs to the Arctic fox genome [2]. The rabies virus isolated from a human rabies case was 100% identical to viruses isolated from two dogs and a mongoose in Nepal [3]. 

Dog bites are the primary source of human infection in all rabies endemic countries and account for 96% of rabies cases in the southeast Asia (SEA) region [4]. Elimination of human rabies is dependent on elimination of dog rabies.

Countries can be categorized depending on rabies status: high, medium, and low rabies endemic countries and rabies-free countries. Maldives, Timor-Leste, and some islands of India are historically free of rabies. Bangladesh, India, and Myanmar are high rabies endemic countries. Bhutan, Nepal, and Sri Lanka are medium rabies endemic countries. Thailand is moving towards low endemic status, but due to increasing rabies incidence Indonesia is moving from a low endemic to a medium rabies endemic country. Rabies is an emerging disease problem on many islands of Indonesia which were previously considered rabies-free. Some countries have a comprehensive rabies control programme but it is a neglected disease problem in others due to competing public health priorities and the complex nature of rabies control activities. 

Prevention of rabies in humans depends on a combination of interventions. These include provision of postexposure prophylaxis (PEP) to exposed patients, preexposure immunization of people at high risk of exposure, control of infection in animal reservoirs, and control of dog populations [5]. Although rabies is preventable, the high cost of vaccines, compounded by the lack of education and awareness about the disease, limits the use of PEP. Recent studies show that most patients were victims of rabies due to negligence, ignorance, or the inadequate availability of primary health care services [4].

Progress in preventing human rabies through control of the disease in its animal reservoir has been slow. This has been due to technical, intersectoral, organizational, and financial obstacles. In addition, there has been a lack of efficient dog rabies control campaigns including humane canine population management [6]. The success and sustainability of dog immunization coverage depends heavily on appropriate management of the dog population. The efforts towards population management are limited and disjointed in most countries. Lethal methods of dog population control have been used in some countries which have been an expensive option. Attempts to control rabies through dog culling have not been sustainable or socially acceptable due to public, religious, and animal welfare concerns. Furthermore, surgical sterilization of dogs in small numbers and at irregular intervals does not yield any long-term benefits in reduction of the population. There are successful programmes of dog population control in limited urban areas coordinated by leading NGOs. However, they are location specific and have not been replicated at rural levels with community participation.

## 2. Burden of Disease

Rabies is a disease of public health and economic importance in southeast Asia. The annual expenditure due to rabies has been estimated to be more than US$ 563 million in Asia [7]. This figure is based on the direct and indirect costs of PEP in humans and costs incurred from dog rabies control efforts. 

Rabies is a disease of poverty, affecting vulnerable populations and children. According to available data, children in the 5–15 year age-group represent about 40% of people exposed to dog bites in rabies endemic areas [4]. The majority of bites that occur in children go unrecognized and unreported and, consequently, exposed children do not receive the benefit of timely and complete courses of postexposure prophylactic treatment [8]. Additionally, paralytic rabies is often misdiagnosed as acute neurological syndrome. Thus, there is the possibility of a disproportionately high number of young children contracting and dying of undiagnosed rabies. 

More than 1.4 billion people are at potential risk of rabies infection in the southeast Asia region.  Table 1 shows countrywise estimates of human rabies and dog bite cases. 

Each year, 21 000–24 000 people die in the SEA Region due to rabies. This accounts for approximately 45% of worldwide human rabies deaths.

Of the estimated 19 million humans bitten by dogs in the SEA Region, it is estimated that at least 4 million receive one or more doses of rabies vaccine [9]. In the majority of countries, the number of patients receiving PEP has steadily increased over time, particularly in urban areas. This is due to improvements in awareness, availability, affordability, and accessibility of safe and effective rabies vaccines particularly in urban areas. Countries are allocating increasing portions of health budgets to procurement of modern rabies vaccines and immunoglobulin to meet the growing demand for PEP.

## 3. Feasibility of Elimination of Human Rabies

The necessary tools and methods for prevention and control of human and canine rabies are available. The proof of the feasibility of elimination of dog-mediated rabies has been demonstrated in countries like Singapore and Malaysia. It is thought that strict enforcement and policies of dog registration, vaccination, and dog population management have made rabies control and eradication effective in these countries. Malaysia borders Thailand, and the concept of an immune belt has been developed by dog licensing and mandatory vaccination of dogs as well as systematic destruction of unvaccinated dogs in a buffer zone to prevent entry of rabies from the northern border. 

Sri Lanka and Thailand have registered a decline in the number of human rabies deaths through implementation of a mass dog vaccination campaign, improved accessibility to PEP, and effective vaccine delivery systems.

Control of rabies through vaccination in the canine population is fundamental to elimination of human rabies. Rabies elimination programmes focused mainly on mass vaccination of dogs are largely justified by the future savings of human rabies prevention programmes. The Pan American Health Organization initiated a regionally coordinated programme for elimination of human rabies transmitted by dogs in 1983. This was mainly based on mass immunization of dogs and has led to a 90% reduction in and elimination of dog rabies from Chile and major urban centers of other Latin American countries [10]. In Mexico, after five years of a nationwide dog vaccination campaign, the number of human rabies deaths was reduced from 60 per year to less than 20 [11]. 

Coordinated mass dog vaccination campaigns will improve herd immunity levels and prevent potential human exposure to rabies but strong political commitment and intensive social mobilization is vital. The active role of the veterinary authority at the national level for animal rabies control is crucial and it is their social responsibility to prevent human rabies through well-planned dog rabies control programmes. There are increasing numbers of international partners for dog rabies control and dog population management in southeast Asia which have been encouraged since the introduction of World Rabies Day in 2007. 

Innovative tools and techniques have been developed and standardized in recent years which will help to improve dog vaccination coverage, accessibility, and affordability of modern rabies vaccine and dog population management. As an adjunct to parenteral immunization, oral rabies vaccines (ORVs) have been extensively tested for efficacy and safety in owned and ownerless dogs. ORV delivery strategies for dogs which cannot be reached by parenteral vaccination have been designed and tested in parts of Asia [12]. However, the cost of oral rabies vaccine for dog immunization is a limiting factor. The use of immunocontraception may be considered, in conjunction with oral and parenteral rabies vaccination, as a complementary tool to reduce the density of dog populations and rabies incidence [13].

## 4. Cost Effectiveness of Intradermal versus Intramuscular Rabies PEP and RIG

India is the only country in the Region producing various types of quality rabies tissue-culture vaccines (TCVs). It is capable of producing 15 million doses of rabies vaccine annually (Personal communication, Dr. RL Ichhpujani), which is sufficient for whole region. Use of RIG in category three bites is limited due to the high cost of HRIG administration. Purified ERIG is now produced in sufficient quantities in India and Thailand and is safe and affordable to use. The availability of highly effective human rabies vaccines and ERIG within the region is important to prevent possible human deaths due to exposure to rabid animals. 

Cost-effective rabies vaccination schedules were introduced in early 1990s. The WHO Expert Committee in 1991 recommended intradermal application of modern rabies vaccines for PEP [14]. Multisite intradermal administration of rabies vaccines reduces the costs of PEP by 60% [15]. The original Thai Red Cross Regimen can be replaced if two doses of vaccine are given on day 0,3,7, and 28 (“2-2-2-0-2” regimen) as per the recommendation of the eighth WHO Expert Consultation [16]. The updated Thai Red Cross Regimen considerably improves compliance rates as patients receive the full course within one month. A research study is ongoing for the administration of a complete PEP intradermal schedule within the period of one week. 

Intradermal rabies vaccination (IDRV) was introduced and widely used in Thailand in the past and has been adapted and promoted in Bangladesh, India, Sri Lanka and other Asian countries. Today, over 95% of patients are provided PEP with an IDRV regimen in Sri Lanka (personal communication, Dr. Omala Wimalaratna). WHO has been providing technical support to other rabies endemic countries to introduce the IDRV regimen. 

The cost of PEP can be reduced dramatically if the intramuscular (IM) regimen is replaced in a progressive manner with the IDRV regimen. The conservative estimate of costing for human rabies prophylaxis, including vaccine and serum application for a period of five years in southeast Asia region, is presented in Tables 2 and 3. Vaccine wastage is expected with IDRV due to the short shelf life of the vaccine after reconstitution and fewer patients at the time of vaccination. While calculating the cost estimates for IDRV, it was estimated that 25% vaccine will be wasted. 

**Figure 1:**
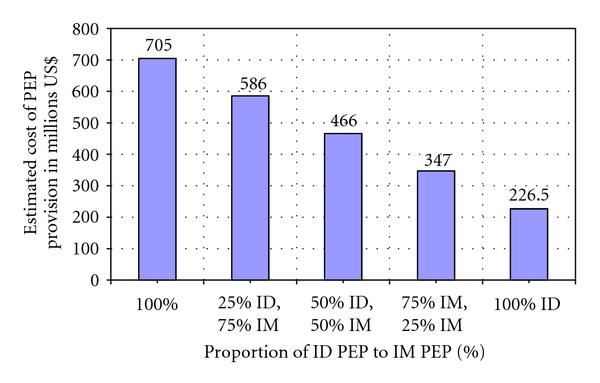
Estimated costs of providing varying proportions of intradermal (ID) versus intramuscular (IM) rabies postexposure prophylaxis (PEP) to 4 million dog bite patients.

Table 2 in the Annex give cost estimates for the use of IM and ID PEP regimes. If only the intramuscular (IM) PEP regime is used over a period of 5 years, the cost estimation is US $705 million. In contrast, if only the intradermal (ID) PEP protocol is practiced the cost estimation will be almost two thirds less at US $226.5 million. This demonstrates the cost-effectiveness of ID versus IM PEP protocols.  Figure 1 illustrates the cost estimate effects of varying the percentage proportion of ID and IM rabies PEP. 

Experience has shown that 25% victims have third category bites that require rabies serum application. Equine rabies immunoglobulin (ERIG) is over 18 times cheaper than human immunoglobulin (HRIG) as shown in Table 3 of Annex 1. Using only HRIG in third-degree bite cases over a five-year period will cost approximately US $1.51 billion, whereas using ERIG over the same period will cost approximately US $57 million.

## 5. Regional Multisectoral Collaboration

Except for island countries, elimination of rabies is not possible without regional cooperation. No single country can maintain rabies-free status unless it is brought under control in neighbouring countries. Regionally coordinated efforts are necessary for elimination of human rabies with consideration of country-specific needs and sociocultural acceptability. WHO launched a regional rabies control project in the 1980s in Asia and many countries developed and strengthened national capacity for rabies surveillance, diagnosis, vaccine production, and dog population management. This encouraged coordination and cooperation between human and animal health sectors for rabies prevention and control at the country level. 

Following the first WHO recommendation in 1984 to replace nerve-tissue vaccines (NTVs), many developing countries of southeast Asia have discontinued the production and use of NTVs for human use [17]. In 2004, the WHO Expert Consultation issued a definitive statement to the effect that NTVs should be discontinued [16]. As a continuous effort of WHO and professional organizations, all countries except Bangladesh and Myanmar have discontinued production and use of the Semple type NTV.

Considering the importance of consolidating achievements in rabies control in Member countries, WHO SEARO came up with the regional strategy for human rabies elimination from southeast Asia in 1998. WHO SEARO organized an intercountry meeting in Colombo, Sri Lanka in 2005 to review the rabies situation in SEA Region and to formulate mechanisms for implementation of the strategy for elimination of rabies [18].

It is important that Member States of the Association of southeast Asian Nations (ASEAN) and the South Asian Association for Regional Cooperation (SAARC) have identified rabies as a priority public health problem. There is also a growing concern and commitment for elimination of human rabies by a number of national governments in the SE Asia Region. There was a SAARC Rabies Meeting in Colombo in 2003 which was attended by government officials of Member countries. The meeting recommended the development of a SAARC strategy for rabies elimination [19]. The ASEAN countries adopted the resolution to prevent and control rabies, with the goal of rabies elimination by year 2020 [20]. The Rabies in Asia Foundation conference held in Hanoi in September 2009 passed resolutions to take several steps to reach the goal of human and dog rabies elimination by 2020. The WHO has been requested to reinforce capabilities to meet the demands from Member States for technical assistance, technology transfer, and the launching of regional initiatives for dog rabies control and elimination in Asia in close collaboration with ASEAN and SAARC [21]. 

The WHO Strategic Framework for the Elimination of Human Rabies Transmitted by Dogs in the South East Asia Region is due for publication in 2011. The document will provide technical guidance for the regional strategy as well as national strategies and act as an advocacy tool to develop consensus among major stakeholders for a comprehensive rabies elimination programme.

## 6. Conclusion

The elimination of human rabies transmitted by dogs is an achievable goal. The initiative has been taken to develop a unique strategic framework for elimination of human rabies transmitted by dogs in consideration of the epidemiological situation, technical feasibility, and the sociocultural context. The cost benefits of using intradermal rabies vaccines and equine immunoglobulin for postexposure prophylaxis has been demonstrated. These techniques must be adopted. The WHO Strategic Framework will be a vital guide for the collaborative, intersectoral approach that is necessary for rabies control. With the adoption of the strategic elements of this document the huge public health and economic burden of human rabies can by eliminated in southeast Asia.

## Figures and Tables

**Table 1:** Distribution per year of human rabies and dog bite cases in countries of the southeast Asia region.

Country	Estimated no. of dog bites	Estimated no. of human rabies cases	Estimated no. of human cases per million population	Source of information
Bangladesh	300,000	2,000–2,500	13	Ministry of Health and Family Welfare, Bangladesh
Bhutan	5000	<10	3	Ministry of Health, Bhutan
DPR Korea	Not available	Not available	Not available	N/A
India	17,400,000	18,000-20,000	18	Assoc. for Prevention and Control of Rabies in India (APCRI)
Indonesia	100,000	150–300	1.3	Ministry of Health, Indonesia
Maldives	0	0	0	N/A
Myanmar	600,000	1000	22	Ministry of Health, Myanmar
Nepal	100,000	<100	4	Ministry of Health and Population, Nepal
Sri Lanka	250,000	<60	3	Public Veterinary Services, Sri Lanka
Thailand	400,000	<25	0	Ministry of Public Health, Thailand
Timor Leste	1,000	0	0	Ministry of Health, Timor-Leste

SE ASIA TOTAL	19,156,000	21,345–23,995		

**Table 2:** Cost estimation of intramuscular (IM) versus intradermal (ID) rabies postexposure prophylaxis (PEP) in the southeast Asia region over a five year period.

Activity	100% IM PEP	100% ID PEP
Estimated number of patients taking PEP (millions)	4	4
Volume of TCV required per person for PEP course (milliliters)	5	1
Total volume of TCV required to achieve 100% coverage (+25% wastage for ID) (millions of mLs)	20	5
Cost per millilitre of TCV, incl. syringe + diluent (US$)	7	9
Cost of TCV vaccine per year (million US$)	140	45
Cost of vaccine for five years (million US$)	700	225
Cost of transportation, storage, etc. (million US$)	5	1.5

Total cost for five years (million US$)	705	226.5

(TCV: tissue culture vaccine. Vaccine costs calculated at current market rates. Transportation and storage costs estimated as 25% of vaccine volume).

**Table 3:** Cost comparison of human rabies immunoglobulin (HRIG) versus equine rabies immunoglobulin (ERIG) for postexposure prophylaxis (PEP) in southeast Asia over a five-year period.

Activity	HRIG	ERIG
Estimated number of patients taking PEP (millions)	4	4
No. of dog bite cases requiring RIG application (millions)	1	1
Quantity of RIG required per patient (millilitres)	5	5
Quantity of RIG required per year (millions of mLs)	5	5
Cost of human RIG per mL (million US$)	60	3
Cost of human RIG for year (million US$)	300	15
Cost of transportation, storage etc. (million US$)	2	1.25

Total cost of RIG for five years (million US$)	1510	81.25

(RIG costs calculated at current market rates. Transportation and storage costs were estimated at 25% of RIG volume).
